# Health-Margin-Aware Load Redistribution from Calibrated Sensor Evidence

**DOI:** 10.3390/s26216933

**Published:** 2026-11-02

**Authors:** Anzuo Jiang, Hongkai Zhu, Heng Li, Wenhan Hou, Haoqiang Sun, Rui Huang

**Affiliations:** School of Computer and Control Engineering, Yantai University, Yantai 264005, China; zhuhongk@s.ytu.edu.cn (H.Z.); liheng@ytu.edu.cn (H.L.); cshwh@ytu.edu.cn (W.H.); sunhaoqiang2004@s.ytu.edu.cn (H.S.); huangrrr@s.ytu.edu.cn (R.H.)

**Keywords:** condition monitoring, posterior calibration, sensor uncertainty, measurement-quality gating, hydraulic systems, fault recovery, load redistribution, constrained action, reproducibility

## Abstract

Condition monitoring can identify abnormal operating states, but a diagnostic posterior does not specify how much load a remaining unit can accept after a dropout. We evaluate a sensor-to-action interface that converts calibrated condition probabilities into a bounded numerical recovery request using 1449 stable cycles from the UCI hydraulic-systems dataset. A four-way split is repeated for 10 seeds. Regularization is selected on validation; temperature, the lower-margin multiplier, the conformal threshold, and the quality-gate threshold are selected on calibration; the test fold is used once for final evaluation. Mean macro-F1 is 0.985 on clean cycles, 0.672 with 10% temporal-SD noise, and 0.766 after VS1 imputation. Relative to posterior-mean requests, the calibration-selected lower margin reduces request exceedance and modeled shortfall under all three inputs. Full recovery increases only under 10% noise (+0.0615); it is essentially unchanged under clean input (−0.0054, descriptive cluster interval −0.0212 to +0.0076) and lower after VS1 imputation (−0.0219, −0.0429 to −0.0019). A fixed κ=0.5 lower margin matches or modestly exceeds the calibration-selected margin in these matched numerical episodes, so calibration selection is not claimed to provide an additional action benefit. In condition-holdout tests under noise, family-level macro-F1 is 0.289–0.354; the worst held-out level is 0.177 and the lowest numerical recovery rate is 0.033. The quality gate abstains on all noisy holdout episodes. At q=0.95, an equal-*z* negative-control gate reduces automatic release from 0.583 to 0.333, with corresponding changes in conditional risk and recovery. Projection enforces the configured envelope, but map, envelope, and delay mismatch can produce true-envelope violations. These results are limited to a numerical model and do not constitute physical, HIL, field, safety, or calibrated-capacity validation.

## 1. Introduction

Sensor-based fault diagnosis has become effective at identifying abnormal operating states, but a diagnostic score is not itself an operating command. Recent reviews identify heterogeneous sensors, measurement quality, process coupling, operating-condition changes, and the difficulty of translating classifier outputs into dependable decisions as persistent problems [1,2,3,4,5]. The distinction is important when a detected degradation is followed by load reassignment after a unit dropout: the estimated state, its uncertainty, the assumed capacity consequence, the coordinator request, and the released command are separate quantities.

Pump and hydraulic-equipment studies illustrate this separation. Recent studies address water-pumping anomalies, mechanism-based diagnosis of electric submersible pumps, small-sample plunger-pump diagnosis, multi-sensor axial-piston-pump diagnosis, centrifugal-pump blockage, and transfer learning [6,7,8,9,10,11]. These studies mainly evaluate diagnosis. Other work considers pump sequencing as an allocation problem within a physical or model-based formulation [12]. Neither line of work specifies how uncertain sensor outputs can be converted into a bounded recovery request when the health-to-capacity relation is declared rather than measured.

Recent work on noise-aware diagnosis, uncertainty calibration, domain adaptation, graph-based process monitoring, and sensor-fault detection provides useful tools for the sensor layer [13,14,15,16,17,18,19,20,21,22,23]. Split conformal prediction has also been used for distribution shift and false-alarm control in industrial processes [16,24]. Its exchangeability-based coverage, however, should not be assumed under the measurement shifts considered here. Other recent studies address feature selection, physics-informed features, edge deployment, and realistic process monitoring [25,26,27,28]. These results motivate a focused interface study rather than another comparison of diagnostic accuracy.

This study does not present a physical capacity estimator or a complete pump-control solution. It examines a bounded sensor-posterior-to-action interface: a probabilistic recognizer supplies a condition posterior; temperature scaling and a declared-label health margin are selected on calibration cycles; a feature-shift score can withhold automatic release; a declared leakage-to-capacity map produces the recovery request; and the request is clipped to the configured envelope at each surviving endpoint. Its specific contributions are as follows:

1. A fully specified interface carrying a calibrated condition posterior through declared health-margin mapping, measurement-quality abstention, capacity-proportional allocation, and local action release.
2. A bounded numerical formulation that separates local configured-envelope projection from model shortfall and from true-envelope or delay mismatch
3. A four-way train/validation/calibration/test evaluation protocol with matched numerical comparisons and targeted audits of operating-condition holdout, map mismatch, gate risk–coverage, allocator resolution, and episode traceability.

**Figure 1:**
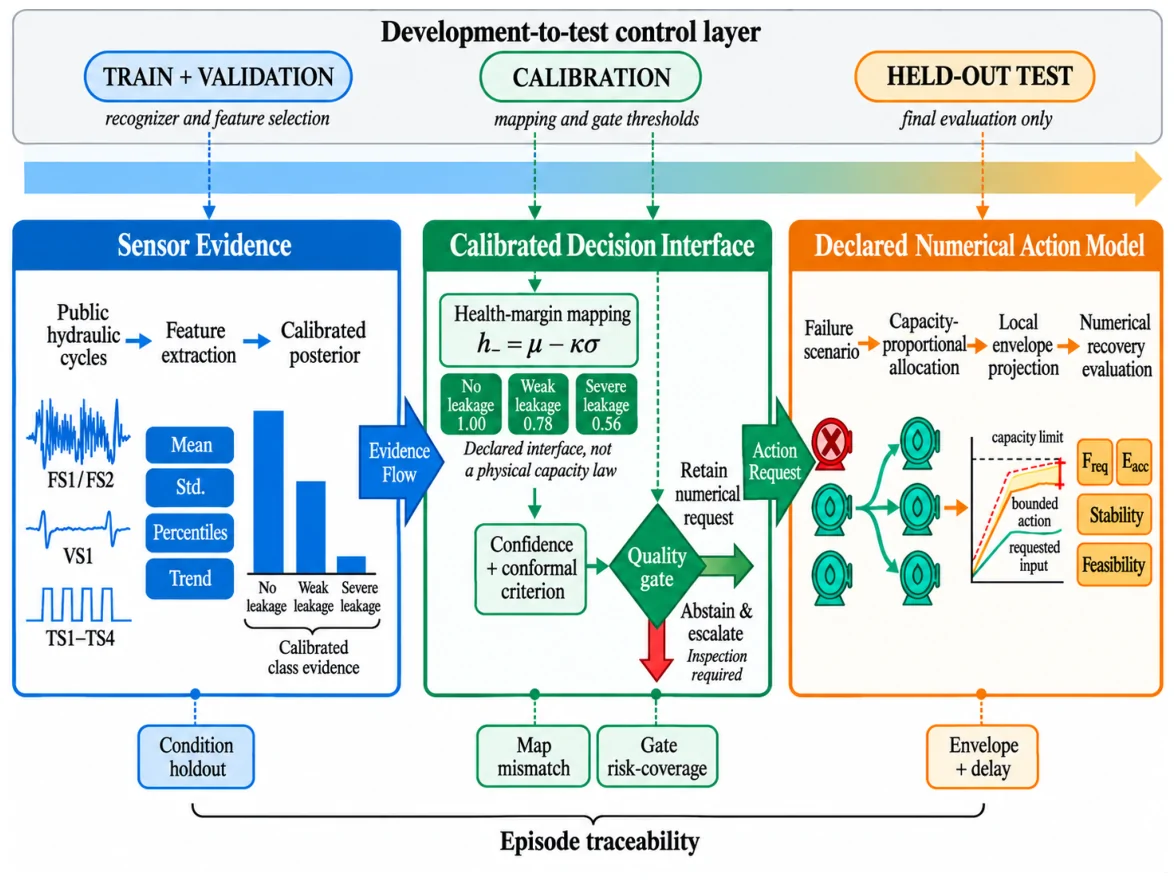
Framework and audit structure of the sensor-to-numerical-action interface. Blue, green, and orange panels denote sensor evidence, the calibrated decision interface, and the declared numerical action model, respectively; arrows indicate evidence and action flow.

Figure 1 summarizes the revised workflow and its audit structure. Public hydraulic cycles are converted into calibrated class probabilities, which enter the declared health-margin mapping and quality gate. A retained request is then allocated, projected to the configured envelope, and evaluated in the numerical action model. The upper layer separates training/validation, calibration, and held-out testing; the lower labels identify the associated checks for condition holdout, map mismatch, gate risk–coverage, envelope–delay stress, and episode traceability. The figure depicts a computational workflow rather than a physical closed-loop or HIL system. Episode definitions and the scope of each reported quantity are given below.

## 2. Problem Formulation and Interface

### 2.1. Declared Capacity and Recovery Allocation

Consider *N* parallel pumps. Pump *i* has requested flow $q_{i}$, rated flow $q_{i}^{\mathrm{rated}}$, and a declared normalized capacity factor $\alpha_{i}$. The factor is a stress-test bridge, not a measured safety limit or calibrated degradation law. The model capacity and available increment are

(1) $$q_{i}^{\max}=q_{i}^{\mathrm{rated}}\alpha_{i},\;m_{i}^{+}=\max\left(0,q_{i}^{\max}-q_{i}\right).$$

For a single failed pump *f*, let $D_{f}=q_{f}$ be the declared lost demand. The numerical coordinator always returns the explicitly feasible recovered increment $R_{f}^{★}$ and allocation $\Delta_{i}$:

(2) $$R_{f}^{★}=\min\left\{D_{f},\sum_{i\neq f}m_{i}^{+}\right\},\;\sum_{i\neq f}\Delta_{i}=R_{f}^{★},\;0\leq \Delta_{i}\leq m_{i}^{+}.$$

The residual $D_{f}-R_{f}^{★}$ is a declared coordinator-visible shortfall. With insufficient stated margin, a derated allocation is returned rather than an infeasible equality or a command outside the declared model envelope. In the primary map, no, weak, and severe leakage correspond to factors 1.00, 0.78, and 0.56, respectively. These values are not interpreted as physical pump-capacity measurements.

The numerical truth and the action interface use separate capacity maps. Let $a_{i}^{\mathrm{true}}$ determine the simulated true envelope and let $a_{i}^{\mathrm{assumed}}$ determine the posterior-to-capacity estimate used in a request. The matched case has $a_{i}^{\mathrm{true}}=a_{i}^{\mathrm{assumed}}=\left[1.00,0.78,0.56\right]$. The mismatch audit evaluates three true maps and five assumed maps, including optimistic, conservative, and crossed weak/severe factors. A favorable result under the matched map therefore does not identify a physical health-to-capacity law.

### 2.2. Posterior-to-Margin Coordination and Local Release

Let $p=(p_{0},p_{1},p_{2})$ be the recognizer posterior for the three declared leakage labels and a=(1.00,0.78,0.56) their declared capacity factors. In each replication, the recognizer is fitted from the training cycles only. A scalar temperature *T* is selected by minimizing negative log likelihood on the disjoint calibration cycles, and ${\tilde{p}}_{k}\propto p_{k}^{1/T}$ is then used on test cycles. The posterior mean and a moment lower bound are

(3) $$\mu_{a}=\sum_{k=0}^{2}{\tilde{p}}_{k}a_{k},\;\sigma_{a}=\sqrt{\sum_{k=0}^{2}{\tilde{p}}_{k}a_{k}^{2}-\mu_{a}^{2}},\;{\underline{a}}_{\kappa }=\max\left\{0.30,\mu_{a}-\kappa \sigma_{a}\right\}.$$

The multiplier κ∈{0,0.25,…,2.00} is selected as the smallest grid value whose calibration-cycle declared-label coverage is at least 0.95. This rule adds conservatism to the declared label map; it is not a confidence interval for measured pump capacity or a physical safety bound. The primary comparison also retains a pre-specified fixed κ=0.5 reference under the same four-way splits and numerical episodes. As a secondary reference, split conformal prediction forms a label set $\Gamma =\{k:1-{\tilde{p}}_{k}\leq \hat{q}\}$ using a 0.05 calibration miscoverage level [16,24] and uses $\min_{k\in \Gamma }a_{k}$ as its declared lower bound. Both references are reported in the matched action comparison; neither is used to claim coverage under shifted measurements.

To make calibration failure operationally visible, a transparent feature-shift score $s\left(x\right)=\sqrt{d^{-1}\sum_{j=1}^{d}{\left(\left(x_{j}-{\bar{x}}_{j}\right)/s_{j}\right)}^{2}}$ is compared with its 95th calibration-cycle percentile. If any of the five selected sensor cycles in a modeled episode exceeds this threshold, no automatic recovery request is released and escalation is logged. Abstention is never counted as recovery, safety, or an accepted command.

For each anticipated single-pump fault, one deterministic capacity-proportional allocation is stored at a fixed estimated-capacity snapshot. The feasible recovered demand in Equation (2) is apportioned according to nonnegative estimated survivor capacities; no general water-filling optimum is implied. After fault confirmation, the record associated with *f* is retrieved. The finite map is constructed before the modeled dropout and is not intended as global pre-enumeration across future health states. Let $r_{i}$ be a retrieved request, ${\bar{c}}_{i}$ the currently configured endpoint envelope, and $c_{i,t}^{\mathrm{true}}$ the simulated true envelope. The released action is

(4) $$u_{i}=\min\left\{\max\left(r_{i},0\right),{\bar{c}}_{i}\right\},\;u_{f}=0.$$

Hence, for finite nonnegative ${\bar{c}}_{i}$, $0\leq u_{i}\leq {\bar{c}}_{i}$ holds by construction. This is an algebraic interface invariant conditional on the configured envelope, not empirical validation that ${\bar{c}}_{i}$ equals $c_{i,t}^{\mathrm{true}}$ or is timely or sufficient for physical safety. The delay/mismatch audit therefore reports configured-envelope exceedance and true-envelope exceedance as separate quantities. A verified command can satisfy the configured projection while exceeding a stale, underestimated, or otherwise mismatched true envelope. Accordingly, zero accepted-envelope exceedance for a verified policy is reported as an invariant check, not as an empirical performance discovery.

Let $R=\sum_{i}r_{i}$ and $U=\sum_{i}u_{i}$. For demand-capped allocation, the projection loss $L_{\mathrm{clip}}=\sum_{i\neq f}\max\left(0,r_{i}-{\bar{c}}_{i}\right)$ yields $U=R-L_{\mathrm{clip}}$ and

(5) $$D-U=\left(D-R\right)+L_{\mathrm{clip}},$$

which separates coordinator-visible insufficiency from local request/envelope mismatch. The projection prevents release beyond its declared interface but does not establish global optimality, functional safety, cybersecurity, or a plant-level recovery guarantee.

Each post-fault numerical episode contains K=450 one-second samples from t=151 to t=600, with demand $d_{t}$, true envelope $c_{i,t}$, request $r_{i,t}$, released command $u_{i,t}$, and simulated delivered flow $y_{i,t}$. The reported episode metrics are

(6) $$\begin{array}{cc} E_{\mathrm{req}} & =K^{-1}\sum_{t}1\left\{\exists i:r_{i,t}>c_{i,t}\right\},\\ E_{\mathrm{acc}} & =K^{-1}\sum_{t}1\left\{\exists i:u_{i,t}>c_{i,t}\right\},\\ S & =\sum_{t}\max\left\{0,d_{t}-\sum_{i}y_{i,t}\right\}, \end{array}$$

and $F=1\{{\bar{e}}_{\mathrm{last}60}\leq 0.05\;\wedge \;E_{\mathrm{acc}}=0\}$, where ${\bar{e}}_{\mathrm{last}60}$ is the mean relative demand error over the final 60 s. Thus, “full recovery” is a declared numerical end-of-episode criterion, *S* is a model-flow×s shortfall integral, and neither quantity is a measured service or safety outcome. Capacity-proportional allocation and elementwise projection are O(N) in the number of active units. For the 24-point demand pattern, the precomputed request table stores 600 float64 values, or 4.8 kB, for five units and 1176 values, or 9.4 kB, for seven units.

## 3. Materials and Methods

### 3.1. Public Sensor Data and Recognizer

The UCI Condition Monitoring of Hydraulic Systems dataset (Dataset 447; UCI Machine Learning Repository, Irvine, CA, USA) contains 2205 60 s cycles; the 1449 stable cycles are retained. The seven channels are FS1 and FS2 (flow), VS1 (vibration), and TS1–TS4 (temperature). For each channel, eight cycle summaries are extracted: mean; standard deviation; minimum; maximum; the 10th, 50th, and 90th percentiles; and the end-minus-start slope. For the primary control, each seed uses a class-stratified four-way split of 724 training, 217 validation, 218 calibration, and 290 test cycles (50/15/15/20%). Validation selects the regularization value. The disjoint calibration fold selects temperature, the declared-label lower-margin multiplier, the conformal quantile, and the feature-shift threshold; the test fold is used only for final evaluation. All diagnosis references use the same 56 summary features and train-plus-validation augmentation. Random forest, RBF least-squares SVM, *k*-nearest neighbors, and nearest centroid were selected to represent an ensemble tree method, a kernel-margin method, a local-neighborhood method, and a low-complexity reference, respectively. Their role is diagnostic context rather than controller selection; the logistic posterior is retained because its calibrated class probabilities enter the declared action mapping.

The primary test set is a held-out-cycle test rather than an unseen-pump test because a pump-asset identifier is not provided in the public data. A post hoc operating-condition check is formed by holding out recorded cooler, valve, or accumulator levels one at a time without retuning the regularization parameter. This intentionally unfavorable test exposes condition-shift limitations.

### 3.2. Measurement-Quality and Recovery Protocols

The controlled measurement-quality suite applies range-clipped zero-mean Gaussian noise from 1% to 10% of the channel median within-cycle standard deviation, positive constant offset from 1% to 10%, and mean-imputed loss of FS1, VS1, or TS1 before summary recomputation. Training-only augmentation is applied only to the training fold at 1%, 2%, and 5% noise. The held-out test set is never used for fitting, classifier selection, temperature selection, margin selection, conformal threshold selection, or shift-threshold selection. These perturbations are synthetic transformations of public measurements, not observed sensor-fault data.

The linked recovery model is composed of five variable-speed pumps connected to a shared header. The 24-point normalized Net3 demand pattern is advanced every 25 s within a 600 s model episode, and a single dropout is imposed at 150 s. Health-scaled quadratic head-flow curves, finite header compliance, speed-rate limits, and declared true envelopes are included. A numerical test bed for posterior mean, posterior lower margin, and local verification is thereby provided; it is not a calibrated digital twin, field trial, HIL platform, or closed-loop pump-controller validation.

The primary matched action comparison uses ten four-way seeded partitions. Six evenly spaced Net3 demand phases and five failed-pump identities give 30 episodes per input condition and seed. Four locally projected strategies are evaluated on the same episodes: posterior mean, fixed κ=0.5 lower margin, calibration-selected lower margin, and split-conformal lower bound. Each episode records five source cycle IDs, the failed-pump identity, the phase index, the capacity-map identifier, and a posterior-update flag. The flag is zero because the posterior is fixed before the episode. Cycle-cluster paired bootstrap intervals are descriptive summaries over unique cycle IDs, not independent-site confidence intervals. A separate 3×3 sensitivity grid is formed from weak-leakage factors {0.70,0.78,0.86} and severe-leakage factors {0.40,0.48,0.56}; every map is monotone and is applied consistently to synthetic true and estimated health. The map-mismatch audit separates true maps from assumed maps and evaluates 15 true/assumed pairs.

### 3.3. Robustness Analyses Across Posterior Backends and Numerical Scenarios

The dependence of the interface direction on the logistic posterior is examined with a fixed 64-tree random forest, a maximum depth of 7, and a minimum leaf size of 4. It is fitted on the same training-only augmented features in each seeded partition. Its temperature and lower-margin multiplier are selected on the same disjoint calibration fold; no test labels are used for classifier or hyperparameter selection. The original five-unit near-balanced header is compared with an unbalanced seven-unit header having rated-flow vector [0.75,0.95,1.05,0.90,1.15,0.85,1.00] and demand fraction 0.60 rather than 0.62. Six demand phases, every single-unit failure identity, and 10% measurement noise are used for each backend/scenario pair. This is a second declared numerical stress scenario, not an independent plant topology or physical validation.

For a gate quantile q∈{0.90,0.95,0.99}, the threshold is the *q*-quantile of calibration-cycle feature-shift scores. The cycle flag rate and five-cycle episode automatic-action availability $A_{q}=M^{-1}\sum_{m=1}^{M}1\left\{\max_{j\in m}s\left(x_{j}\right)\leq h_{q}\right\}$ are reported under clean input, 10% noise, 5% positive offset, FS1 loss, and VS1 loss. In a separate equal-calibration-budget comparison, the 0.95 quantile is set independently for feature distance, posterior uncertainty $1-\max_{k}{\tilde{p}}_{k}$, and normalized posterior entropy. Each comparator therefore has an approximately 5% nominal calibration-cycle flag budget before shifted test inputs are inspected. For each known synthetic transformation versus its matched clean cycles, fixed-threshold balanced flag discrimination $B=\frac{1}{2}\left[\mathrm{Pr}\left(s>h|\mathrm{transformed}\right)+\mathrm{Pr}\left(s\leq h|\mathrm{clean}\right)\right]$ and threshold-free score AUC are additionally reported; these are controlled transformation-separation summaries, not OOD or anomaly-detection performance. Under the same 10%-noise lower-margin estimate, online exact capacity-proportional allocation, capped equal sharing, and finite precomputed tables with declared health-bin widths 0.025, 0.05, and 0.10 are compared; posterior-mean and lower-margin estimates are also compared under the identical online allocator. The same local projection is used for every comparator. The declared full-recovery criterion is recalculated at final 60 s relative-error thresholds 0.02, 0.05, and 0.10 without test-based threshold selection. Timing is obtained from repeated local measurements on the recorded workstation and is retained as a software microbenchmark, not as a PLC, network, or actuator latency measurement. The data sources, derived quantities, and interpretation limits are summarized in Table 1. For the condition-holdout analysis, cooler, valve, and accumulator levels are held out one level at a time; the held-out level is excluded from model fitting, calibration, and threshold selection. The full chain is evaluated under clean input, 10% temporal-SD noise, and VS1 imputation. The level-wise summary retains all 11 held-out levels and reports the minimum macro-F1, maximum request rate, minimum recovery rate, and maximum shortfall for each holdout family and sensor condition. In the envelope-delay audit, configured scale (0.9/1.0/1.1), envelope error (0/5%), and command delay (0/5/20 s) are crossed; released commands are checked against both configured and true envelopes. The gate risk–coverage analysis reports the automatic-action availability and conditional risk at feature-distance quantiles 0.90, 0.95, and 0.99, with abstained episodes kept explicit. A negative-control gate combines feature distance, predictive uncertainty, and normalized entropy after calibration-fold *z*-scoring with equal weights; it is not tuned as a replacement. OpenAI Codex (a GPT-5-based coding agent) was used for code debugging, reproducible table and figure preparation, and language editing. It was not used to generate data, select analyses, or interpret results; the authors reviewed the code, outputs, and manuscript.

## 4. Results

### 4.1. Recognition Context and Operating-Condition Limitations

**Figure 2:**
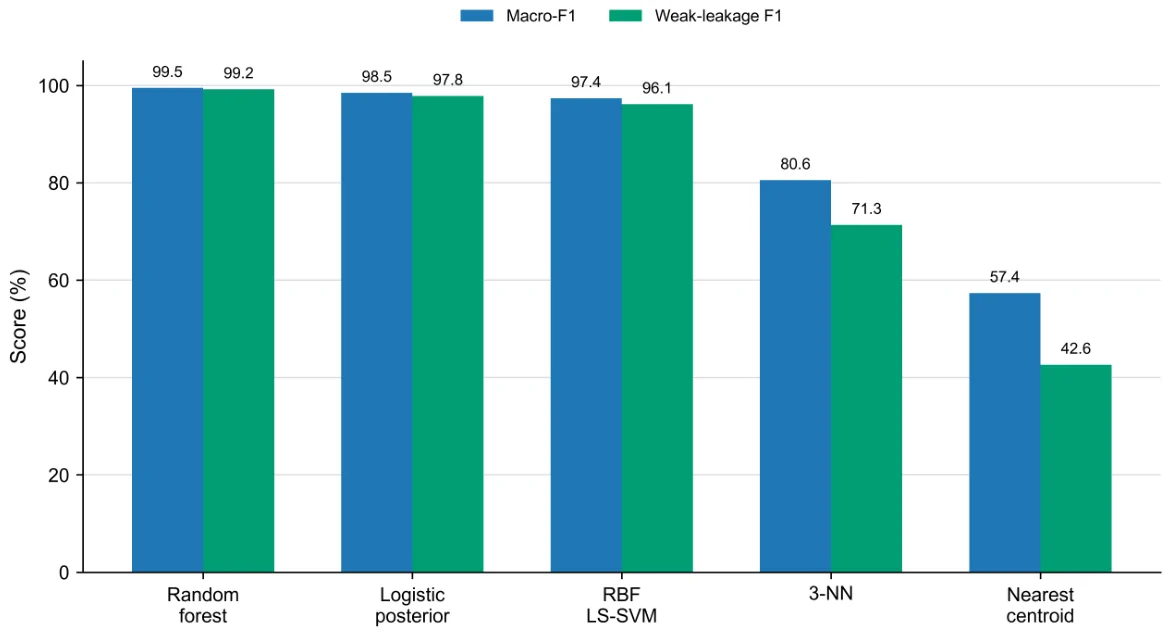
Diagnosis-only reference models.

Across the ten four-way held-out test partitions, the multinomial logistic posterior has mean accuracy 0.985 and macro-F1 0.985. The diagnosis-only random-forest, RBF LS-SVM, 3-NN, and nearest-centroid references have mean macro-F1 values of 0.995, 0.974, 0.806, and 0.574, respectively. Table 2 and Figure 2 provide this diagnosis context. Both table and figure report percentage-based scores over the same ten test partitions; no recovery outcome is represented. The logistic posterior is retained for its defined downstream semantics rather than as a diagnostic state-of-the-art choice.

Operating-condition holdout is less favorable than the random split. The minimum accuracy is 0.965 for valve levels and 0.878 for accumulator levels, whereas cooler-level holdout falls to 0.635 and weak-leakage recall is 0.006 for cooler level 3. This prevents interpreting random-split accuracy as invariant across operating conditions or sites.

The full-pipeline condition holdout shows the limitation beyond accuracy. Three cooler levels, four valve levels, and four accumulator levels are held out without refitting calibration or gate thresholds. Mean macro-F1 is 0.7713/0.9855/0.9498 on clean input for cooler/valve/accumulator holdouts, and falls to 0.2892/0.3536/0.2924 under 10% temporal-SD noise. Under the same noise, lower-margin verified request rates are 0.6228, 0.7056, and 0.7417, while numerical full-recovery rates are 0.4667, 0.1917, and 0.2333. The quality-gated policy abstains on all noisy holdout episodes; these episodes are not counted as recoveries.

Family means conceal level-specific failures. The individual-level audit finds the lowest macro-F1 at the valve level-90 holdout under 10% noise (0.1772), the largest lower-verify request rate at the accumulator level-90 holdout under 10% noise (0.8130), and a lowest numerical full-recovery rate of 0.0333 at valve level 73 and accumulator level 100 under 10% noise. The largest lower-verify shortfall is 183.23 model-flow×s at accumulator level 90 under 10% noise. These extrema are reported as operating-condition diagnostics, not as unseen-pump or site-generalization estimates.

**Figure 3:**
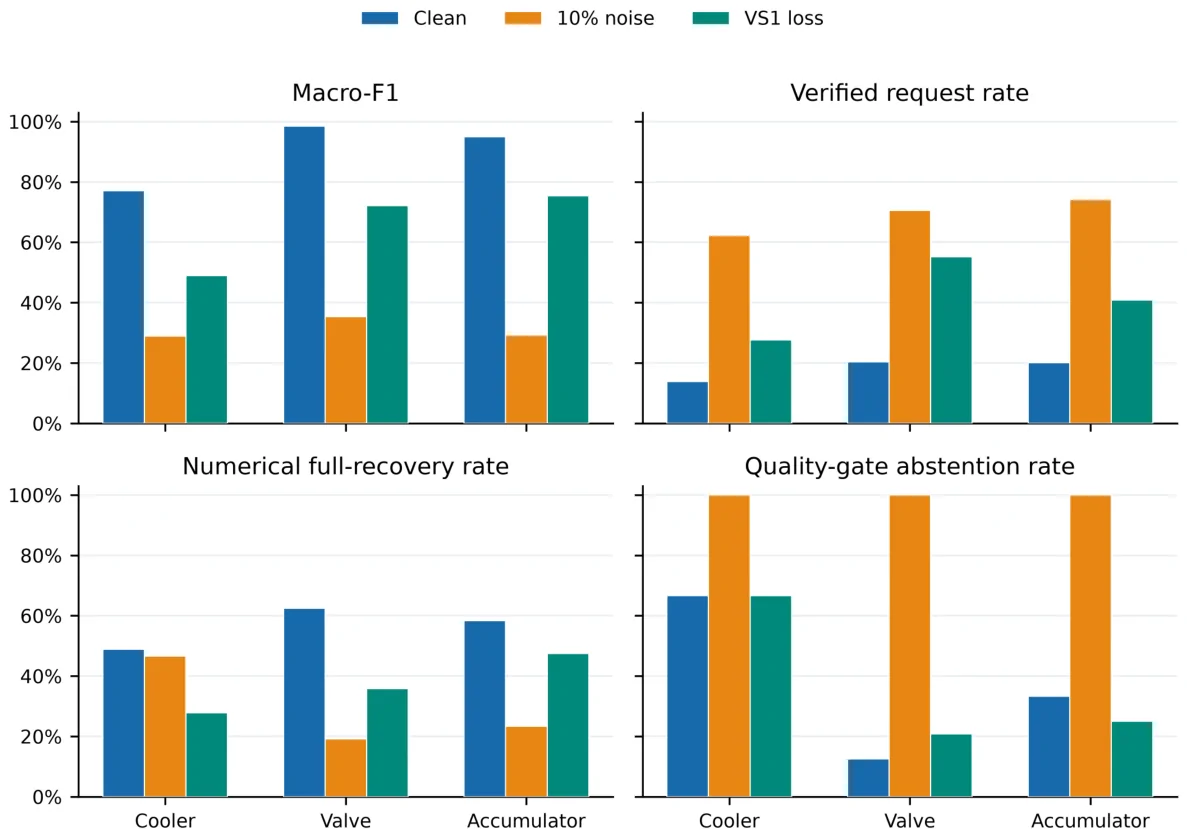
Condition-holdout full-pipeline audit across recorded operating-level families.

Figure 3 provides a compact view of the family-level condition-holdout audit in Table 3 and Table 4. The display separates recognizer performance from numerical request, recovery, and abstention outcomes, so the degradation under the declared perturbations can be inspected without collapsing these quantities into one score.

### 4.2. Four-Way Lower-Margin Comparison and Fixed Baseline

**Figure 4:**
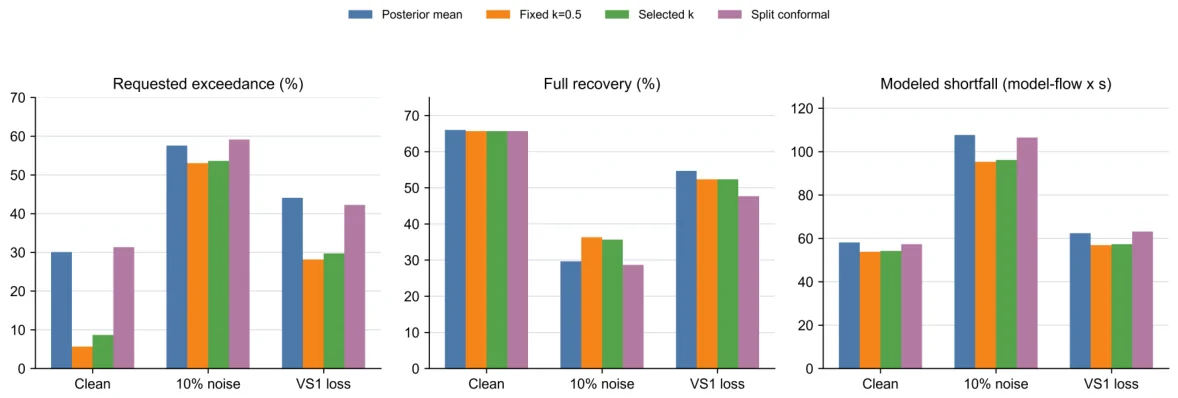
Matched four-way action baselines. Each panel summarizes the same numerical episodes used in Table 5.

Table 5 reports the four-way comparison on the same 30 numerical episodes per input condition and seed. All four strategies use local projection, so accepted-envelope exceedance is zero throughout. The calibration-selected multiplier equals 0.5 in eight of ten seeds and 0.25 in the other two. Relative to the fixed κ=0.5 reference, calibration selection increases requested exceedance by 0.0298, 0.0056, and 0.0154 under clean input, 10% noise, and VS1 loss, respectively, and increases modeled shortfall by 0.33, 0.87, and 0.49 model-flow×s. Full recovery is identical under clean input and VS1 loss and is 0.0067 lower under noise. Thus, the present numerical study does not support an additional action benefit from calibration selection over the fixed 0.5 margin. Figure 4 provides the corresponding panel summaries.

### 4.3. Capacity-Map and Numerical-Scenario Sensitivity

**Figure 5:**
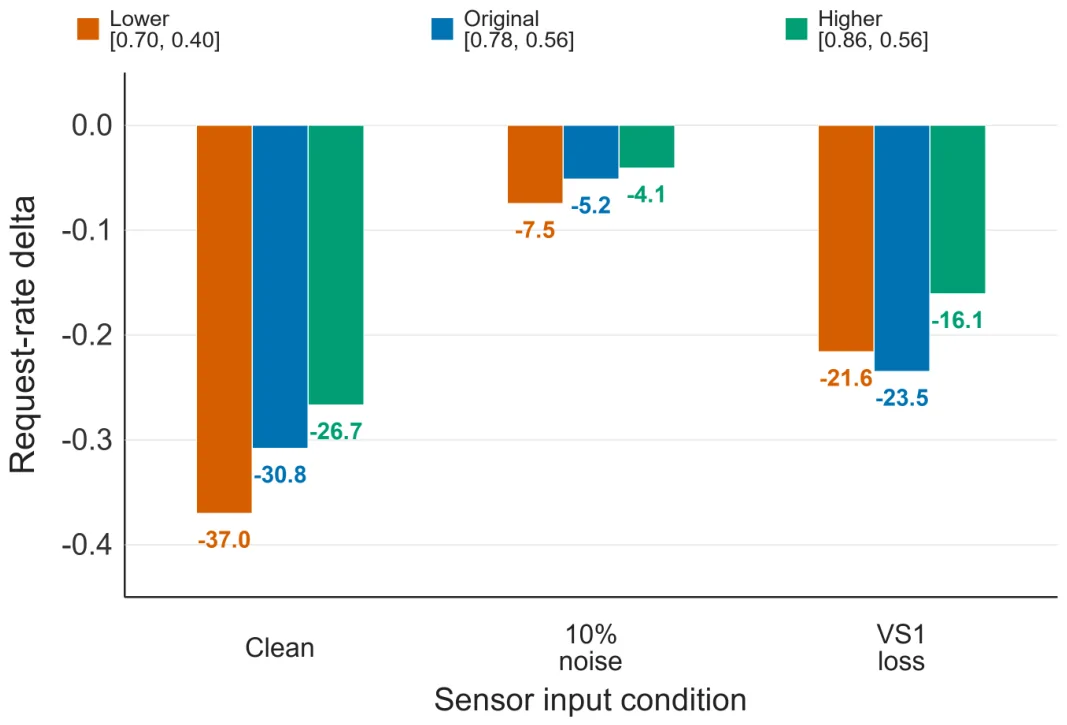
Requested -exceedance sensitivity for three representative maps from the full 3×3 monotone grid.

The matched true-map=assumed-map nine-cell grid is a separate numerical sensitivity analysis, not an estimate of the primary four-way calibration effect. It shows lower model-requested exceedance and shortfall across its declared maps. The separate true/assumed mismatch audit in Table 6 is more informative about map dependence; full recovery is not treated as uniformly robust once true and assumed maps are separated. Figure 5 shows the requested-envelope-exceedance sensitivity for representative maps.

The true/assumed map mismatch audit is more conservative than the matched-map grid. Across 15 true/assumed pairs, the lower-minus-mean request change remains non-positive and the shortfall change remains favorable in all three input conditions, but full recovery is not uniform. Under 10% noise, request changes range from −0.1565 to −0.0019, shortfall changes from −25.50 to −6.00 model-flow×s, and recovery changes from −0.0667 to +0.1083; 14 of 15 map pairs have a favorable recovery direction.

**Figure 6:**
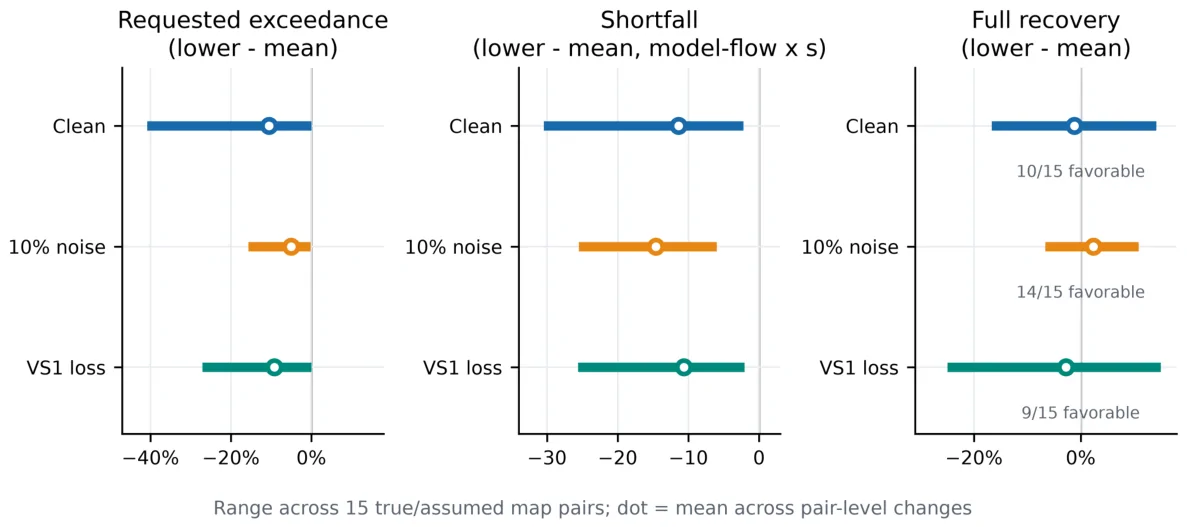
True/assumed capacity-map mismatch audit.

Figure 6 visualizes the range of pair-level changes in Table 7. Negative values favor the calibrated lower-margin request for requested exceedance and shortfall; recovery is deliberately shown separately because its direction is not uniform.

The configured/true-envelope and command-delay stress test separates the projection invariant from physical-envelope compliance. With configured scale 1.0 and zero envelope error, configured-envelope exceedance is zero for released commands, but true-envelope violation is nonzero under both noisy inputs and all tested delays. Under 10% noise, true-envelope violation is 0.6123, 0.3889, and 0.6913 at 0, 5, and 20 s delay, with shortfall 88.40, 74.95, and 123.00 model-flow×s. Under VS1 loss, the corresponding violation rates are 0.4667, 0.3912, and 0.4857, with shortfall 70.83, 72.88, and 91.51. The full grid also exposes configured-envelope violations from stale delayed commands (maximum 0.82), so local projection alone cannot certify a true or time-aligned envelope.

**Figure 7:**
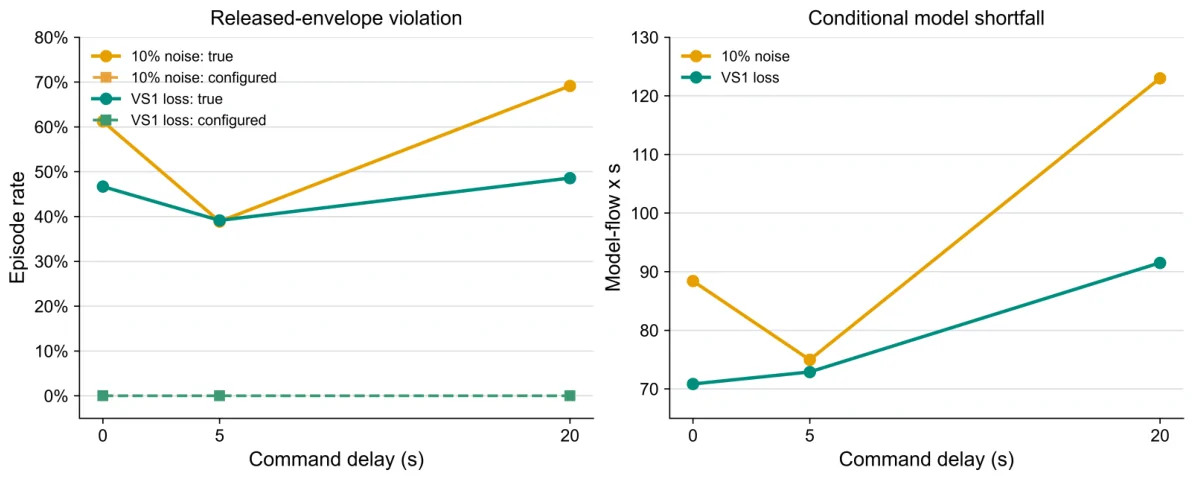
Envelope and delay stress audit at configured scale 1.0 and zero envelope error. The configured traces for 10% noise and VS1 loss both equal 0% and therefore overlap on the zero baseline.

Figure 7 gives the corresponding zero-error, configured-scale-one slice of Table 8. It makes the separation between configured projection and the unavailable true envelope visible across the tested delays.

### 4.4. Posterior-Backend and Numerical-Scenario Transfer

Two questions not addressed by the primary logistic/five-unit experiment are examined: whether the interface direction depends on the posterior backend and whether it persists in a numerically distinct unit-count/rated-flow scenario. Calibration-only posterior preparation is reported in Table 9. Stronger held-out diagnosis under 10% synthetic noise is obtained with the fixed random forest, but no diagnostic state-of-the-art claim is made. In that table, clean and 10%-noise coverage denote declared-label coverage of the selected moment lower margin; no physical-capacity coverage is represented. Declared lower-bound coverage is lost under the shift by both backends; the need for measurement-quality escalation is therefore retained.

**Figure 8:**
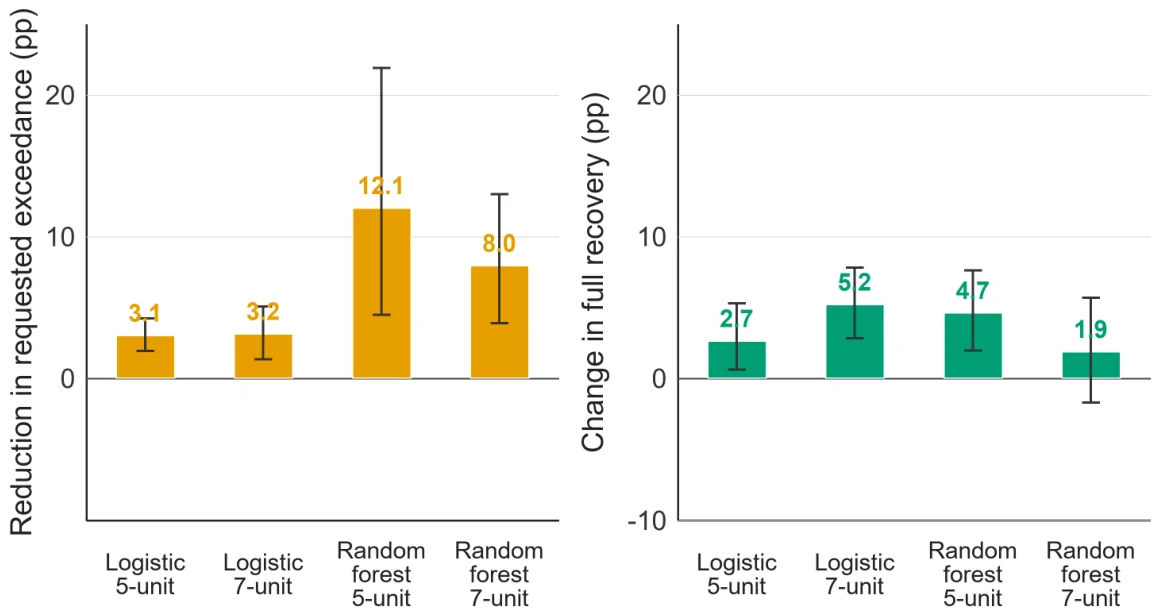
Posterior -backend and numerical-scenario transfer under 10% synthetic noise.

Table 10 shows lower requested-envelope exceedance in all four backend/scenario averages under 10% noise. For logistic posteriors, reductions of 3.1 pp in the five-unit case and 3.2 pp in the seven-unit case are obtained; the corresponding full-recovery changes are +2.7 pp and +5.2 pp. For random forests, request reductions of 12.1 pp and 8.0 pp are obtained. The bars and whiskers in Figure 8 reproduce these lower-minus-mean averages and their descriptive repeated-split ranges; they are not population confidence intervals. The seven-unit random-forest full-recovery difference is only +1.9 pp and its descriptive interval crosses zero; a universal recovery improvement is therefore not supported, even though request reduction and lower shortfall remain directionally stable.

On the recorded workstation, a 128-cycle batch is processed in 45.6 μs for the logistic posterior and 26.3 ms for the fixed random forest; one five-unit capacity-proportional-allocation-plus-projection calculation requires 7.0 μs. These measurements describe local software overhead under the supplied implementation only. Acquisition, PLC scans, communication, fault confirmation, and actuator latency are not included.

### 4.5. Calibration Separation, Shift Sensitivity, and Gated Abstention

The calibration-control experiment separates the 1449 stable cycles into 724 training, 217 validation, 218 calibration, and 290 held-out test cycles in each of ten replications. Regularization is selected on validation only; temperature, the lower-margin multiplier, the conformal threshold, and the gate threshold are selected on the disjoint calibration fold. Across seeds, the mean selected temperature is 0.82 (range 0.50–1.10), the lower-margin multiplier is 0.50 (0.25–0.75), and declared-label moment coverage is 0.978 on clean input, 0.604 under 10% noise, and 0.900 under VS1 imputation. These are declared-label diagnostics, not measured capacity intervals.

**Figure 9:**
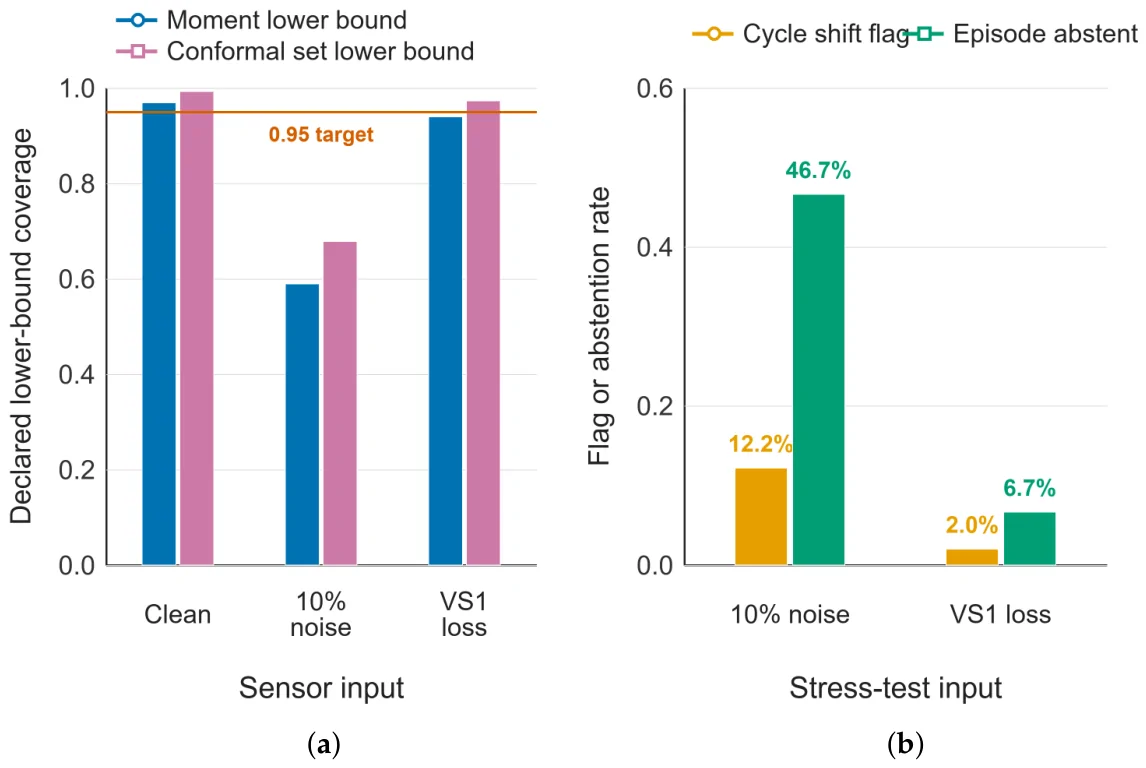
Split -safe declared lower-bound coverage and transparent measurement-quality gate. In (**a**), blue and pink bars denote moment and conformal lower-bound coverage; in (**b**), orange and green bars denote the cycle shift-flag and episode-abstention rates, respectively.

**Figure 10:**
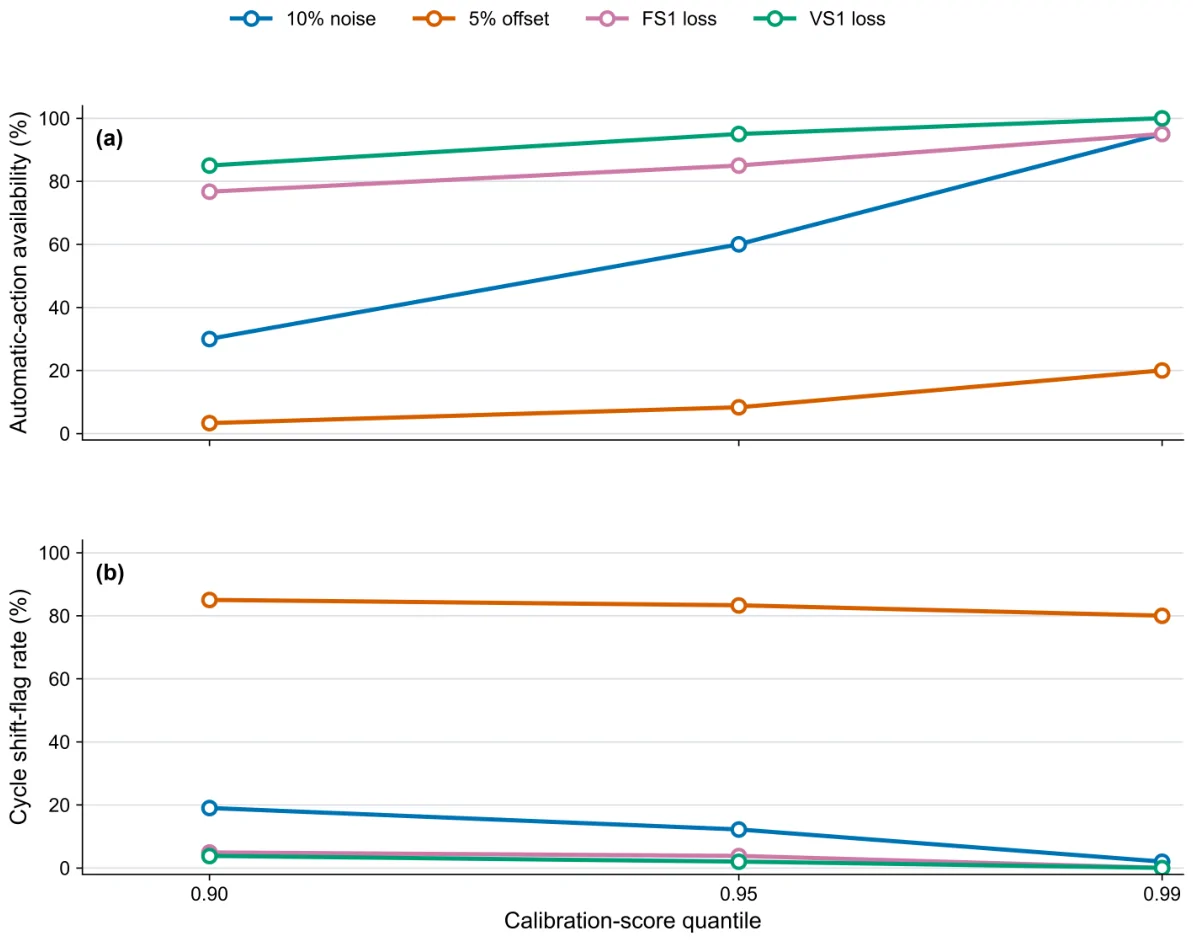
Gate -threshold results for the six-phase robustness event set: (**a**) five-cycle automatic-action availability; (**b**) cycle shift-flag rate.

Table 11 and Figure 9 summarize the transfer results. Mean macro-F1 is 0.985 on clean held-out cycles, 0.672 under 10% synthetic noise, and 0.766 under VS1 imputation. Mean ECE increases to 0.229 under noise, while moment and conformal declared-label coverage fall to 0.604 and 0.698. Thus, neither post hoc temperature scaling nor the conformal reference transfers nominal calibration to these measurement shifts. Calibration on nominal cycles alone does not justify automatic release. Figure 10 reports the corresponding automatic-action availability and cycle shift-flag rates across gate thresholds.

At the numerical-action layer, the cycle-cluster paired comparison of calibration-selected lower margin versus posterior mean changes requested exceedance by −0.0392 [descriptive cluster interval −0.0461 to −0.0327], modeled shortfall by −11.5363 [−12.3185 to −10.7635] model-flow×s, and full recovery by +0.0615 [+0.0476 to +0.0762] under 10% noise. The recovery result does not transfer across inputs: it is −0.0054 [−0.0212 to +0.0076] under clean input and −0.0219 [−0.0429 to −0.0019] after VS1 imputation. Thus, recovery improves only in the tested noise condition, is not clearly changed for clean input, and is lower for VS1 loss. Request exceedance and modeled shortfall remain lower in all three input conditions. The intervals are descriptive cycle-cluster resamples over overlapping seeded partitions, not independent-site confidence intervals.

Removing local verification from the same lower-margin request increases accepted over-envelope exceedance by 0.5359 under 10% noise and by 0.2970 under VS1 loss; full recovery changes by −0.1933 and −0.3267, respectively. The clean-input increase in accepted exceedance is 0.0865. A lower shortfall for the unverified request cannot be treated as a benefit because it is obtained by releasing commands beyond the configured envelope. This numerical ablation supports local release verification within the interface, but it is not a physical guard-function test.

The feature-shift score flags 11.5% of 10%-noise cycles and 2.8% of VS1-loss cycles on average. The corresponding 76.9% and 90.7% automatic-release rates are calculated over 58 consecutive five-cycle windows per held-out test fold; they are therefore not directly comparable with the 30 demand-phase/failure numerical episodes per seed used in the gate tables below. In the harmonized numerical event set, feature-distance automatic-release rates under 10% noise are 31.7/58.3/95.0% at quantiles 0.90/0.95/0.99. Abstained episodes are excluded from conditional quantities and are not counted as recoveries. These thresholds are not OOD, safety, or operational-policy certificates.

### 4.6. Recovery-Criterion Sensitivity, Coordinator Baselines, and Equal-Budget Gate Comparison

The declared five-percent final 60 s relative-error threshold is not a test-selected operating requirement. Dependence of the lower-margin comparison on this reporting threshold is assessed in Table 12 by recalculating the same ten-seed, 10%-noise, locally projected numerical episodes at 2%, 5%, and 10%. The aggregate lower-minus-mean full-recovery difference remains positive at all three thresholds, but the seed-level pattern is sparse: at 2%, one seed is negative and five tie; at 5%, six seeds tie; and at 10%, four seeds tie. The seed-sign entries list positive, negative, and tied seed-wise differences in that order. A directional aggregate statement, rather than a universal per-split service advantage, is supported by this threshold check.

Table 13 separates the local implementation property of precomputation from numerical service ranking. Its final column reports the modeled shortfall integral in model-flow×s, followed after the slash by the median local Python 3.12.13 calculation time in μs; it is not a composite performance score. Under the same calibrated lower-margin estimate and local projection, online exact capacity-proportional allocation reduces requested exceedance by 2.63 pp relative to the 0.05-binned precomputed table. The descriptive interval extends from 0.94 to 4.52 pp. Shortfall is reduced by 0.24 model-flow×s, with an interval from 0.07 to 0.40. The 5%-criterion recovery change is only +0.67 pp, with an interval from 0.00 to 1.67 pp, and no conclusive service improvement is inferred. Worse requested exceedance and shortfall are obtained with capped equal sharing. Numerical optimality is not claimed for the precomputed lookup; its retained benefit is a smaller local software operation: 0.70 μs versus 6.46 μs for online exact allocation in the recorded-workstation microbenchmark. The stated timing boundary remains applicable.

The feature-distance risk–coverage curve makes the abstention trade-off explicit under 10% noise. Table 14 now uses one harmonized set of 30 numerical episodes per seed for both the feature-distance curve and the equal-*z* negative control. At calibration quantiles 0.90, 0.95, and 0.99, feature-distance automatic-action availability is 0.3167, 0.5833, and 0.9500, respectively. The q=0.95 feature-distance row is shared with the negative-control comparison rather than being recomputed on a different episode set. Conditional quantities exclude abstained episodes and do not establish a safety or OOD guarantee.

**Figure 11:**
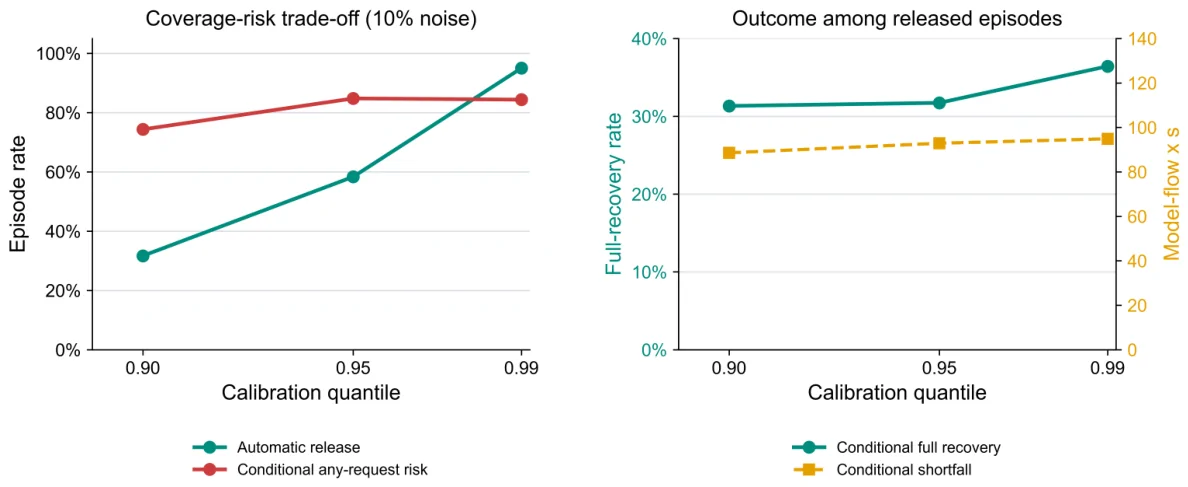
Feature-distance gate risk–coverage summary under 10% temporal-SD noise. Conditional risk, recovery, and shortfall are reported only for released numerical episodes.

Figure 11 plots the retain/abstain trade-off summarized in Table 14. All conditional quantities are evaluated only among released numerical episodes, while availability is reported over the full modeled episode set.

As a negative control, the equal-weight combination of calibration-fold *z*-scored feature distance, predictive uncertainty, and normalized predictive entropy is evaluated on the same q=0.95 rows in Table 14. It releases 0.3333 rather than 0.5833 of the numerical episodes, and its conditional any-request rate, recovery, and shortfall are 0.6933, 0.4467, and 77.65, respectively, versus 0.8477, 0.3173, and 92.87 for feature distance. The comparison is therefore an availability–risk trade-off, not a gate ranking.

**Figure 12:**
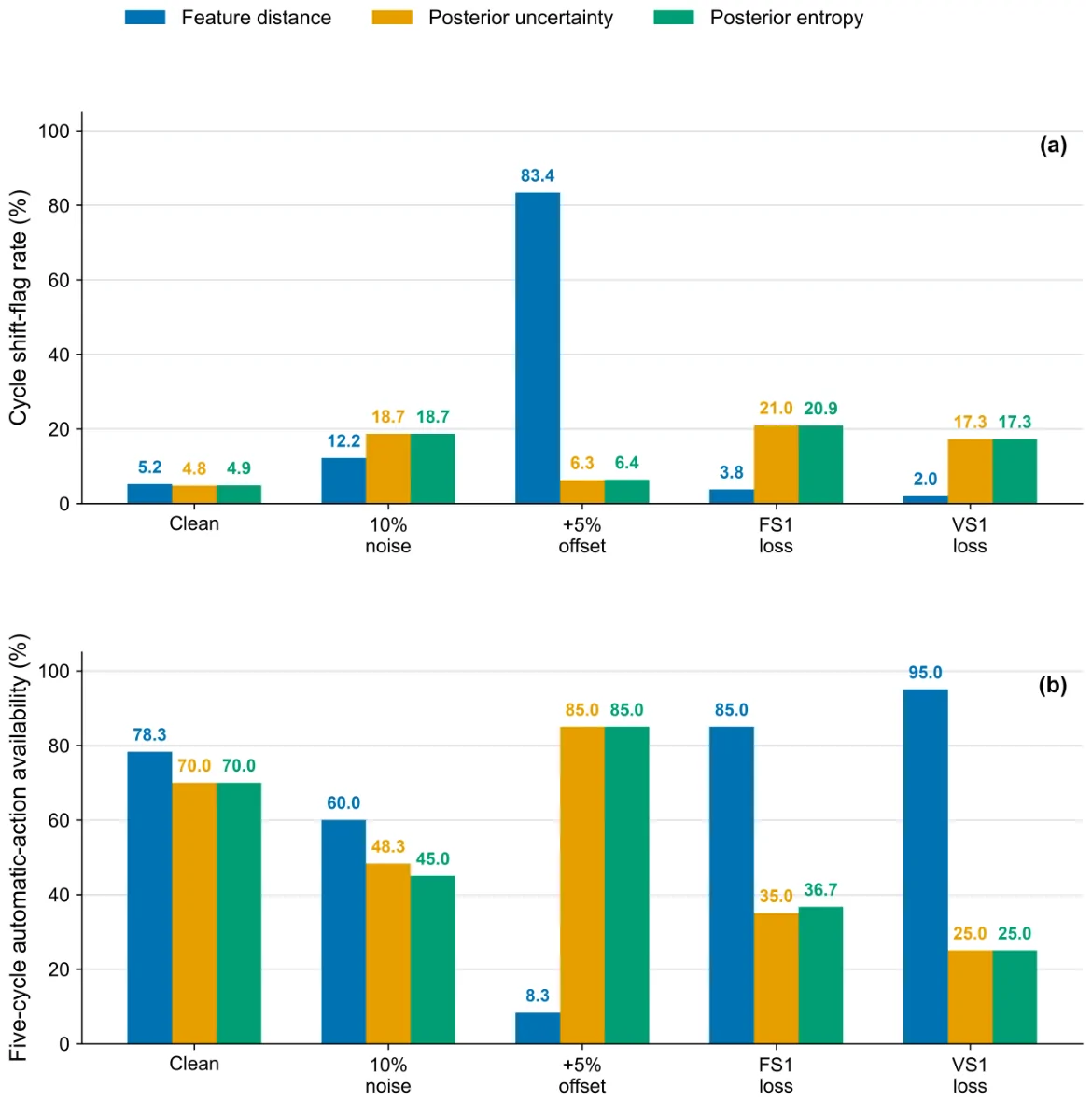
Equal-calibration-budget gate comparison: (**a**) cycle shift-flag rate; (**b**) five-cycle automatic-action availability.

Uniform dominance is not established for the feature-distance gate. Figure 12 compares cycle shift-flag rate and five-cycle automatic-action availability after individual 0.95 calibration-score quantiles are assigned to feature distance, posterior uncertainty, and posterior entropy. Under a 5% positive offset, 83.4% of cycles are flagged by feature distance and 8.3% of five-cycle episodes are released, whereas approximately 6.3% are flagged and 85.0% are released by the two posterior-score gates. Under 10% noise and imputed FS1/VS1 loss, more cycles are flagged and fewer actions are released by posterior scores. The result is condition-dependent escalation behavior, not a detector ranking, OOD guarantee, or operational policy.

### 4.7. Matched Allocator Isolation, Finite-Table Resolution, and Known-Transformation Separation

The matched allocator analysis resolves an ambiguity in Table 13: a posterior-mean precomputed reference cannot isolate the health-margin effect from the allocation operator. Under the same online exact capacity-proportional operator, the calibrated lower margin reduces requested-envelope exceedance by 5.35 pp, with a descriptive repeated-split interval from 3.35 to 7.65 pp. Modeled shortfall is reduced by 8.04 model-flow×s, with an interval from 5.62 to 10.52, and final relative-flow error is reduced by 0.0056, with an interval from 0.0040 to 0.0073. These three directions are favorable in all ten split-specific aggregates. The declared 5% full-recovery difference is +3.33 pp with six ties and a descriptive range including zero; a general recovery improvement is therefore not inferred. The exact sign-flip diagnostic for request and shortfall is 0.0029 in this repeated-split protocol and is retained as an algorithmic sensitivity check rather than population inference because the UCI test sets overlap across seeds.

**Figure 13:**
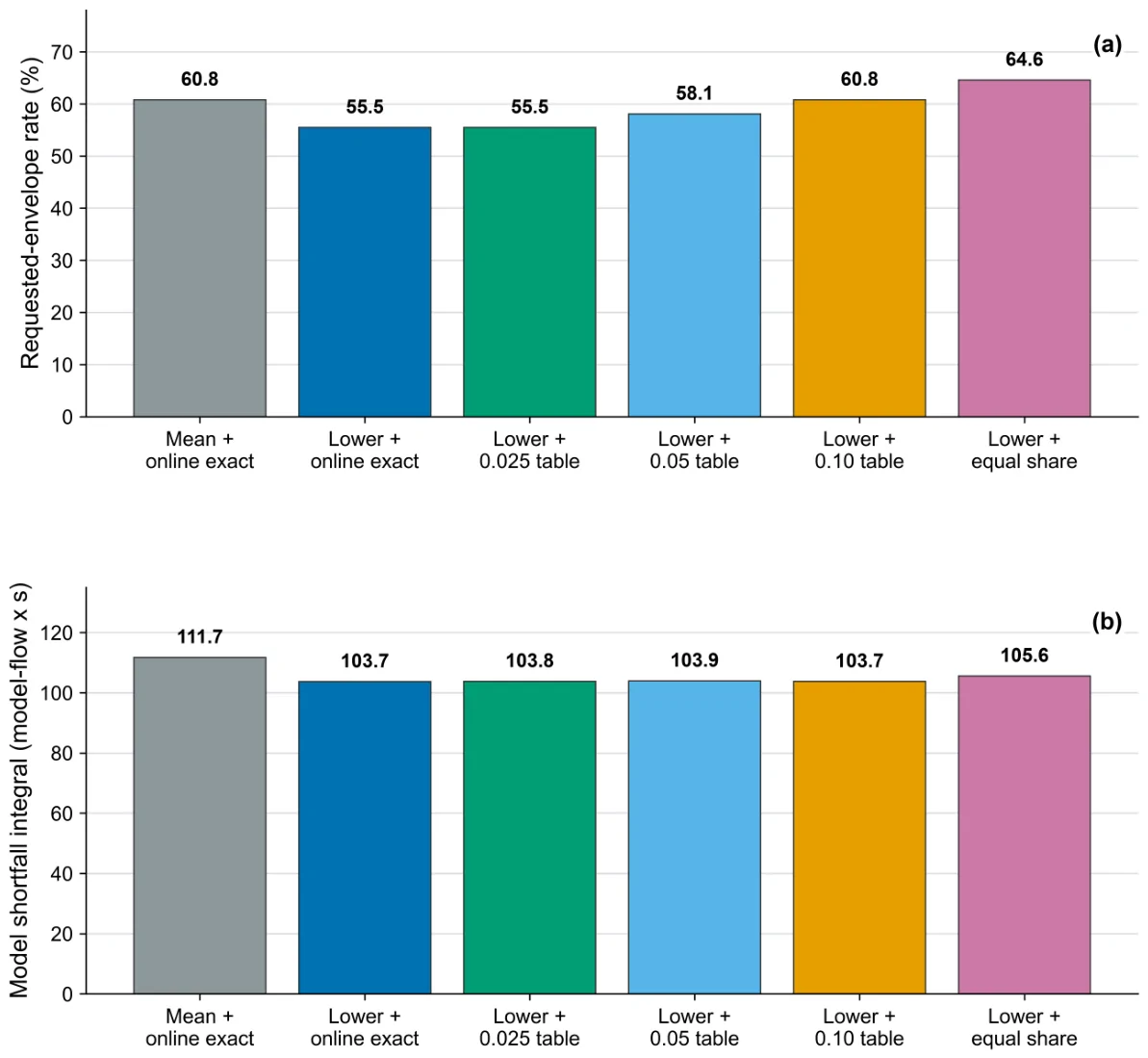
Matched allocator comparisons: (**a**) requested-envelope-exceedance rate; (**b**) modeled-shortfall integral.

The finite-table resolution trade-off is reported in Table 15 and Figure 13. The 0.025-binned lower-margin table differs from online exact allocation by only +0.02 pp in requested exceedance and +0.10 model-flow×s in shortfall on average, with no recovery-rate difference. The 0.05-binned table adds +2.63 pp requested exceedance and +0.24 model-flow×s shortfall; the 0.10-binned table is less consistent. A narrow implementation conclusion is supported: fine-resolution precomputation can closely approximate online allocation while requiring a smaller local software operation. A globally optimal, physical, or real-time controller is not established. Median local Python microbenchmark times are 0.72, 0.70, and 0.72 μs for the 0.025, 0.05, and 0.10 tables, respectively, versus 6.46 μs online and 18.54 μs for capped equal sharing; sensing, communication, PLC, and actuator timing are excluded.

**Figure 14:**
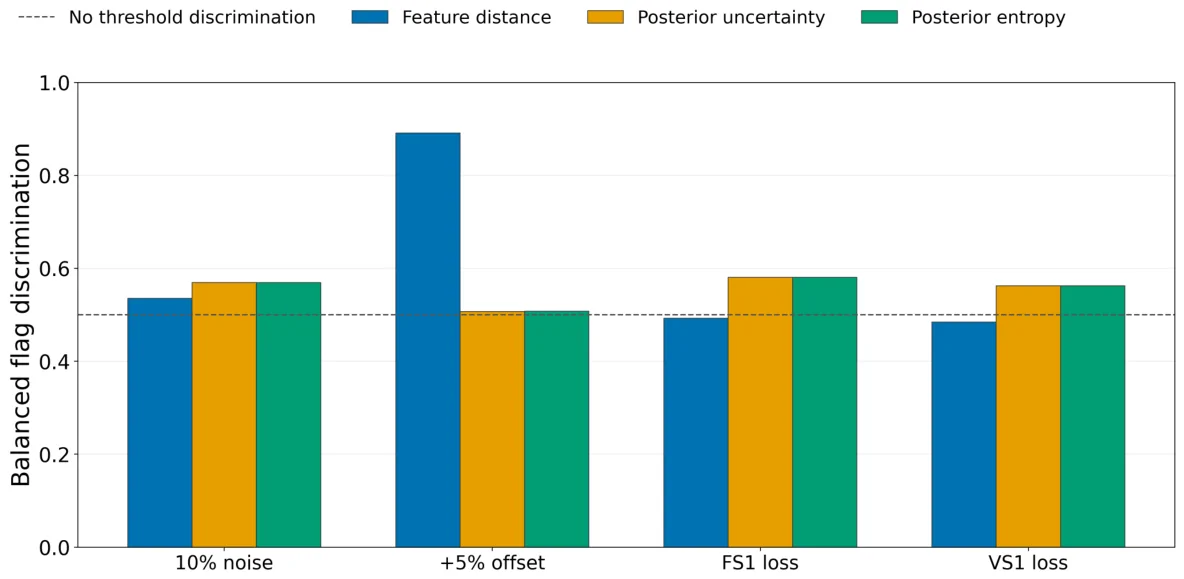
Equal-calibration-budget fixed-threshold separation of known synthetic transformations from matched clean UCI cycles.

The gate comparison is reported without relabeling it as OOD detection. In Figure 14, each declared synthetic transformation is treated as a known positive condition and clean held-out cycles as the matched negative condition, while every score retains its independently set 0.95 calibration-fold threshold. The 0.50 reference denotes no fixed-threshold separation, and values below it indicate separation in the opposite direction from the specified score rule. Balanced threshold discrimination is 0.89 for feature distance under the 5% offset but falls below 0.50 for imputed FS1/VS1 loss; values of about 0.58 for FS1 loss and 0.56 for VS1 loss are obtained with posterior uncertainty and entropy, whereas values near 0.51 are obtained for offset. These results quantify transformation-specific separation and the corresponding automatic-action availability; they do not validate a general anomaly detector, OOD certificate, or operational escalation policy.

The episode traceability audit records 900 paired episodes built from 269 unique source cycle IDs. Each record contains five cycle IDs, the failed-pump identity, the demand phase, the declared map identifier, and a fixed-posterior flag. A source cycle appears 16.73 times on average in the trace and in 1.07 seeded partitions. Cycle-cluster bootstrap results retain the request and shortfall directions, whereas the recovery direction depends on the input condition. The corresponding cycle-cluster intervals are reported in Table 16.

## 5. Discussion

Taken together, the results support a bounded interface claim. With validation, calibration, and test evaluation separated, the calibration-selected lower margin reduces requested-envelope exceedance and modeled shortfall relative to posterior mean under the tested inputs. Its recovery result is explicitly condition-dependent: recovery is higher only under 10% noise, unchanged within the descriptive interval under clean input, and lower after VS1 imputation. The matched fixed-margin comparison further shows that calibration selection does not provide an additional action advantage over κ=0.5 in these numerical episodes. The true/assumed map audit shows why this direction should not be interpreted as identification of a physical capacity law: shortfall is stable over the declared grid, but recovery changes sign for some mismatches. The condition-holdout pipeline also shows strong operating-condition sensitivity, especially for cooler and accumulator levels under synthetic noise. The configured projection is a local algebraic invariant, but the envelope-delay audit shows that it cannot certify a true or time-aligned physical envelope. The quality gate is therefore an abstention heuristic, not an OOD detector or safety policy.

The level-wise holdout audit gives the same qualification in concrete terms: valve/noise macro-F1 reaches 0.1772, accumulator/noise request exceedance reaches 0.8130, and numerical recovery falls to 0.0333 at individual held-out levels. The equal-*z* negative control shows that a different transparent score can lower conditional risk by releasing fewer episodes. Gate selection is therefore a risk–coverage choice, not a basis for claiming a universally superior detector.

The study remains narrower than a controller-validation study. The UCI cycles are controlled-rig measurements without asset or site identifiers; the ten seeded test partitions overlap; and the 900 traced episodes reuse source cycles. Noise, bias, channel loss, and delay are controlled transformations or numerical assumptions rather than observed sensor faults or measured communication delays. Calibration, lower-margin coverage, and gate scores are evaluated against declared labels and maps, not measured pump capacity. The numerical model contains no calibrated pump curves, fluid transients, sensor or actuator dynamics, PLC timing, multi-pump faults, or physical guard behavior. A group-separated physical or HIL study is therefore still needed, with measured health-to-capacity curves, independent pump or site identities, time-stamped sensor and actuator delays, and pre-registered safety-relevant acceptance criteria. More advanced representation, adaptation, federated, and physics-informed methods remain useful directions for the sensor layer [15,17,18,21,22,23,27,28].

## 6. Conclusions

This study evaluates a bounded sensor-to-numerical-action interface. The four-way calibration protocol, condition-holdout audit, true/assumed map comparison, configured/true envelope stress test, gate risk–coverage analysis, equal-*z* negative control, and episode traceability record set the statistical and physical limits of the study. In the declared numerical model, lower margins reduce request exceedance and shortfall relative to posterior mean, but recovery is higher only under the tested noise condition and is lower after VS1 imputation. A fixed κ=0.5 lower margin matches or modestly exceeds the calibration-selected margin in the matched comparisons, so calibration selection is not presented as an additional performance gain. Local verification guarantees the configured projection, and quality gating trades automatic availability against abstention. The results do not establish a field controller, HIL system, physical-capacity law, or functional-safety mechanism. Future work should replace the declared map with measured pump curves, collect group-separated asset and site data, model sensor-to-actuator timing, and validate the complete chain on a physical or HIL platform before safety-relevant use.

## Figures and Tables

**Table 1:** Data sources, derived quantities, and interpretation limits.

Component	Data or Setting	Interpretation Limit
Condition state	Public multichannel hydraulic cycles	Held-out-cycle diagnostic performance
Measurement stress	Synthetic noise, offset, and channel loss	Controlled robustness stress test
Capacity map	Declared monotone label-to-factor map	Sensitivity analysis, not calibration
Posterior calibration	Disjoint calibration-cycle temperature and margin selection	Declared-label uncertainty, not physical-capacity coverage
Quality gate	Calibration-quantile feature-shift score and automatic-action abstention	Escalation diagnostic, not anomaly/OOD or safety certification
Recovery model	Five-pump shared-header numerical model	Numerical model comparison
Endpoint rule	Equation (4)	Configured-envelope compliance by construction

**Table 2:** Diagnosis -only reference comparison over ten four-way held-out UCI test partitions.

Model	Accuracy	Macro-F1	Weak-Leakage F1	Interface Role
Multinomial logistic	0.985	0.985	0.978	Interface posterior
Random forest	0.995	0.995	0.992	Nonlinear ensemble reference
RBF LS-SVM	0.974	0.974	0.961	Kernel-margin reference
3-nearest neighbors	0.807	0.806	0.713	Local-neighborhood reference
Nearest centroid	0.581	0.574	0.426	Low-complexity reference

**Table 3:** Condition-holdout full-pipeline audit across recorded operating levels.

Holdout	Input	Macro-F1	Gate Flag	Lower Request	Lower Recovery	Quality-Gate Abstain
Cooler	Clean	0.7713	0.6667	0.1389	0.4889	0.6667
Cooler	10% noise	0.2892	0.9972	0.6228	0.4667	1.0000
Cooler	VS1 loss	0.4900	0.6667	0.2759	0.2778	0.6667
Valve	Clean	0.9855	0.0450	0.2037	0.6250	0.1250
Valve	10% noise	0.3536	0.9952	0.7056	0.1917	1.0000
Valve	VS1 loss	0.7220	0.0249	0.5519	0.3583	0.2083
Accumulator	Clean	0.9498	0.0793	0.2014	0.5833	0.3333
Accumulator	10% noise	0.2924	0.9959	0.7417	0.2333	1.0000
Accumulator	VS1 loss	0.7543	0.0433	0.4088	0.4750	0.2500

**Table 4:** Individual-level extrema in the condition-holdout full-pipeline audit.

Holdout	Input	Minimum Macro-F1	Maximum Lower Request	Minimum Lower Recovery	Maximum Lower Shortfall
Cooler	Clean	0.5305 (3)	0.4000 (20)	0.3000 (3)	61.15 (20)
Cooler	10% noise	0.2715 (100)	0.7667 (20)	0.4333 (3)	97.93 (20)
Cooler	VS1 loss	0.2222 (100)	0.7056 (20)	0.1000 (100)	104.48 (20)
Valve	Clean	0.9753 (100)	0.2926 (100)	0.5333 (73)	63.90 (73)
Valve	10% noise	0.1772 (90)	0.7907 (90)	0.0333 (73)	133.66 (73)
Valve	VS1 loss	0.6699 (80)	0.6537 (100)	0.1000 (90)	90.81 (73)
Accumulator	Clean	0.8718 (130)	0.2241 (130)	0.4333 (100)	80.63 (100)
Accumulator	10% noise	0.2159 (115)	0.8130 (90)	0.0333 (100)	183.23 (90)
Accumulator	VS1 loss	0.6804 (130)	0.6333 (100)	0.3000 (100)	94.13 (100)

**Table 5:** Four-way matched action comparison for posterior mean, fixed and calibration-selected lower margins, and split conformal.

Input	Strategy	Req. Exceed.	Full Recovery	Shortfall
Clean	Posterior mean	0.3007	0.6600	58.14
Clean	Fixed κ=0.5	0.0567	0.6567	53.87
Clean	Calibration-selected κ	0.0865	0.6567	54.20
Clean	Split conformal	0.3131	0.6567	57.35
10% noise	Posterior mean	0.5756	0.2967	107.68
10% noise	Fixed κ=0.5	0.5304	0.3633	95.34
10% noise	Calibration-selected κ	0.5359	0.3567	96.22
10% noise	Split conformal	0.5911	0.2867	106.49
VS1 loss	Posterior mean	0.4409	0.5467	62.39
VS1 loss	Fixed κ=0.5	0.2817	0.5233	56.86
VS1 loss	Calibration-selected κ	0.2970	0.5233	57.35
VS1 loss	Split conformal	0.4226	0.4767	63.16

**Table 6:** Full 3×3 monotone label-to-capacity sensitivity grid.

Sensor Input	Grid	Request-Rate Delta	Full-Recovery Delta	Interpretation
Clean	9 maps	−41.3 to −18.5 pp	+0.8 to +9.2 pp	Uniform request reduction; recovery positive
10% noise	9 maps	−7.5 to −4.1 pp	+4.2 to +22.5 pp	Uniform request reduction; recovery positive
VS1 loss	9 maps	−28.3 to −16.1 pp	−3.3 to +8.3 pp	Request reduction; recovery not uniform

**Table 7:** True/assumed capacity-map mismatch audit. Deltas are calibrated-lower minus posterior-mean numerical outcomes over 15 map pairs.

Input	Request Delta Range	Shortfall Delta Range	Recovery Delta Range	Favorable Recovery Pairs
Clean	−0.4083 to 0.0000	−30.48 to −2.24	−0.1667 to +0.1417	10/15
10% noise	−0.1565 to −0.0019	−25.50 to −6.00	−0.0667 to +0.1083	14/15
VS1 loss	−0.2708 to 0.0000	−25.64 to −2.06	−0.2500 to +0.1500	9/15

**Table 8:** Configured-envelope versus true-envelope behavior under delay mismatch at configured scale 1.0 and zero envelope error.

Input	Delay (s)	True Violation	Configured Violation	Shortfall
10% noise	0	0.6123	0.0000	88.40
10% noise	5	0.3889	0.0000	74.95
10% noise	20	0.6913	0.0000	123.00
VS1 loss	0	0.4667	0.0000	70.83
VS1 loss	5	0.3912	0.0000	72.88
VS1 loss	20	0.4857	0.0000	91.51

**Table 9:** Calibration-only posterior preparation in the backend/scenario robustness analysis.

Posterior	Clean Macro-F1	10% Noise Macro-F1	Clean Coverage	10% Noise Coverage	Selected κ
Logistic	0.982	0.650	0.969	0.590	0.375
Random forest	0.993	0.961	0.962	0.717	0.275

**Table 10:** Backend/scenario action transfer under 10% synthetic noise.

Posterior	Numerical Scenario	Request Delta	Full-Recovery Delta	Outcome
Logistic	Five-unit reference	−3.1 pp [−4.3, −2.0]	+2.7 pp [+0.7, +5.3]	Both directions favorable
Logistic	Seven-unit stress	−3.2 pp [−5.1, −1.4]	+5.2 pp [+2.9, +7.9]	Both directions favorable
Random forest	Five-unit reference	−12.1 pp [−21.9, −4.5]	+4.7 pp [+2.0, +7.7]	Both directions favorable
Random forest	Seven-unit stress	−8.0 pp [−13.0, −3.9]	+1.9 pp [−1.7, +5.7]	Recovery not conclusive

**Table 11:** Four-way calibration separation and measurement-quality stress test.

Test Input	Macro-F1	NLL	ECE	M. Cov.	C. Cov.	Shift Flag
Clean	0.985	0.106	0.032	0.978	0.994	4.9%
10% noise	0.672	1.297	0.229	0.604	0.698	11.5%
VS1 loss	0.766	0.705	0.130	0.900	0.953	2.8%

**Table 12:** Sensitivity of the declared numerical full-recovery criterion to the final 60 s relative-error threshold under 10% synthetic noise.

Threshold	Mean Policy	Lower Margin	Lower–Mean	95% Range	Seed Signs
2%	10.7%	15.7%	+5.0 pp	[+0.3, +11.0] pp	4+/1−/5=
5%	28.0%	30.7%	+2.7 pp	[+0.3, +5.7] pp	4+/0−/6=
10%	65.7%	71.0%	+5.3 pp	[+2.0, +9.0] pp	6+/0−/4=

**Table 13:** Equal-input coordinator comparison under 10% synthetic noise.

Coordinator	Req. Exceed.	Accepted Exceed.	Full Recovery	Shortfall/ Timing
Posterior mean + precomputed allocation	61.2%	0.0%	28.0%	111.98/0.70 μs
Lower margin + precomputed allocation	58.1%	0.0%	30.7%	103.93/0.70 μs
Lower margin + online exact allocation	55.5%	0.0%	31.3%	103.69/6.46 μs
Lower margin + capped equal share	64.6%	0.0%	31.0%	105.57/18.54 μs

**Table 14:** Harmonized gate risk–coverage and negative-control comparison under 10% temporal-SD noise.

Gate and Quantile	Auto Release	Abstention	Conditional Any Request Exceed.	Conditional Recovery	Conditional Shortfall
Feature distance, 0.90	0.3167	0.6833	0.7433	0.3133	88.56
Feature distance, 0.95	0.5833	0.4167	0.8477	0.3173	92.87
Feature distance, 0.99	0.9500	0.0500	0.8437	0.3643	94.88
Equal-*z* combination, 0.95	0.3333	0.6667	0.6933	0.4467	77.65

**Table 15:** Matched allocator-resolution check under 10% synthetic noise.

Estimator and Allocation	Req. Exceed.	Shortfall	Full Recovery
Posterior mean + online exact	60.8%	111.74	28.0%
Lower margin + online exact	55.5%	103.69	31.3%
Lower margin + 0.025 table	55.5%	103.79	31.3%
Lower margin + 0.05 table	58.1%	103.93	30.7%
Lower margin + 0.10 table	60.8%	103.74	31.3%
Lower margin + capped equal share	64.6%	105.57	31.0%

**Table 16:** Cycle -cluster paired bootstrap for calibrated lower minus posterior mean. Intervals are descriptive resamples over unique cycle IDs.

Input	Request Delta [95% Interval]	Shortfall Delta [95% Interval]	Recovery Delta [95% Interval]
Clean	−0.2119 [−0.2403,−0.1844]	−3.8817 [−4.3388,−3.4314]	−0.0054 [−0.0212,+0.0076]
10% noise	−0.0392 [−0.0461,−0.0327]	−11.5363 [−12.3185,−10.7635]	+0.0615 [+0.0476,+0.0762]
VS1 loss	−0.1436 [−0.1684,−0.1200]	−4.9707 [−5.6586,−4.3078]	−0.0219 [−0.0429,−0.0019]

## Data Availability

The UCI Condition Monitoring of Hydraulic Systems data are publicly available at https://archive.ics.uci.edu/dataset/447/condition-monitoring-of-hydraulic-systems (accessed on 28 October 2026).The EPANET Net3 input is publicly available at https://github.com/OpenWaterAnalytics/EPANET (accessed on 28 October 2026). The Supplementary Materials archive supplies the frozen scripts, derived diagnosis-reference outputs, four-way calibration-separation outputs, condition-holdout full-pipeline and level-wise worst-case outputs, capacity-map mismatch results, envelope/delay stress tests, feature-distance and equal-z gate summaries, cycle traceability records, validators, and SHA-256 manifests.

## Supplementary Materials

The following supporting information can be downloaded at https://www.mdpi.com/article/10.3390/s1010000/s1. The following supporting information is available with this manuscript: Supplementary Code and Results Revision.zip, containing the public-input provenance, frozen Python code, four-way calibration-separation outputs, condition-holdout full-pipeline and level-wise worst-case outputs, true/assumed capacity-map mismatch results, configured/true envelope and delay stress tests, feature-distance risk–coverage and equal-z negative-control summaries, cycle-level episode traceability, figures, verification reports, and SHA-256 manifests.
